# Dynamic changes in Bach1 expression in the kidney of rhabdomyolysis-associated acute kidney injury

**DOI:** 10.1371/journal.pone.0180934

**Published:** 2017-07-13

**Authors:** Masakazu Yamaoka, Hiroko Shimizu, Toru Takahashi, Emiko Omori, Hiroshi Morimatsu

**Affiliations:** 1 Department of Anesthesiology and Resuscitology, Okayama University Medical School, Okayama, Japan; 2 Department of Faculty of Health and Welfare Science, Okayama Prefectural University, Okayama, Japan; Universidade de Sao Paulo, BRAZIL

## Abstract

Free heme, a pro-oxidant released from myoglobin, is thought to contribute to the pathogenesis of rhabdomyolysis-associated acute kidney injury (RM-AKI), because renal overexpression of heme oxygenase-1 (HO-1), the rate-limiting enzyme in heme catabolism, confers protection against RM-AKI. BTB and CNC homology 1 (Bach1) is a heme-responsive transcription factor that represses HO-1. Here, we examined the changes with time in the gene expression of Bach1, HO-1, and δ-aminolevulinate synthase (ALAS1, a heme biosynthetic enzyme) in the rat kidney using an RM-AKI model induced by the injection of 50% glycerol (10 mL/kg body weight) into bilateral limbs. We also examined the protein expression of Bach1 in the nucleus and cytosol, and HO-1 in the rat kidney. Glycerol treatment induced significant elevation of serum creatinine kinase and aspartate aminotransferase levels followed by the marked elevation of serum blood urea nitrogen and creatinine levels, which caused serious damage to renal tubules. Following glycerol treatment, HO-1 mRNA and protein levels were significantly up-regulated, while ALAS1 mRNA expression was down-regulated, suggesting an increase in the free renal heme concentration. The Bach1 mRNA level was drastically increased 3 h after glycerol treatment, and the increased level was maintained for 12 h. Nuclear Bach1 protein levels were significantly decreased 3 h after treatment. Conversely, cytosolic Bach1 protein levels abruptly increased after 6 h. In conclusion, we demonstrate the dynamic changes in Bach1 expression in a rat model of RM-AKI. Our findings suggest that the increase in Bach1 mRNA and cytosolic Bach1 protein expression may reflect de novo Bach1 protein synthesis to compensate for the depletion of nuclear Bach1 protein caused by the induction of HO-1 by free heme.

## Introduction

Rhabdomyolysis is associated with extensive muscle injury that is accompanied by the release of myoglobin, which causes severe oxidative damage, ultimately leading to acute kidney injury. The heme moiety of myoglobin can initiate lipid peroxidation due to redox cycling of the heme group from ferrous to ferric and then to ferryl oxidation states to cause renal injury [1]. Heme oxygenase-1 (HO-1), which is the rate-limiting enzyme in the catabolism of heme and the primary inducer of HO-1, is induced in the kidney in a rodent model of rhabdomyolysis-associated acute kidney injury (RM-AKI). HO-1 protects against renal oxidative damage, suggesting that in addition to the heme moiety of myoglobin, free heme released from myoglobin contributes to the pathogenesis of RM-AKI [2, 3].

BTB and CNC homology 1 (Bach1) is a heme-responsive transcription factor that represses HO-1. Under baseline conditions, Bach1 binds to small Maf proteins to form a heterodimer that in turn, binds to the Maf recognition element (MARE) in the promoter region of *Ho-1* to repress transcription. In the presence of excess free heme, Bach1-Maf is released from MARE, allowing transcriptional activation of *Ho-1* by nuclear factor (erythroid-derived 2)-like 2 (Nrf2)–Maf heterodimers [4–7].

We hypothesized therefore that Bach1 expression is dynamically influenced by changes in heme metabolism in the kidney of RM-AKI. To test this hypothesis, here we used a rat model of RM-AKI produced by the injection of glycerol to the hind paw and examined the kinetics of gene expression of Bach1, HO-1, and ALAS1 (the rate-limiting enzyme in heme biosynthesis), and Bach1 protein expression in the cytosolic and nuclear fractions of kidney cells after glycerol treatment.

## Materials and methods

### Animals

Animal experiments were approved by the Animal Use and Care Committee of Okayama University Medical School (OKU-2014411). Care and handling of the animals were conducted in accordance with National Institutes of Health guidelines for Animal Research. Male Sprague–Dawley rats weighing 210−250 g were purchased from Clea Japan, Inc. (Tokyo, Japan). Rats were housed in a temperature-controlled room (25°C) with 12 h/12 h light/dark cycles and were allowed free access to water and chow until the start of the experiments.

### Experimental design

The rats were deprived of drinking water for 24 h. They were then randomly divided into the groups as follows: 1. glycerol-treated (glycerol group), 2. saline-treated (saline group), and 3. untreated (untreated control group). The animals in the glycerol group were injected intramuscularly into the bilateral limbs with 50% glycerol (10 mL/kg; Ishizu Seiyaku Ltd, Osaka, Japan) dissolved in an equal volume of saline. The animals in the saline group were intramuscularly (i.m.) injected with the same volume of physiological saline. The conscious rats were placed in a cage after injection and were again allowed free access to chow and tap water. After the desired period after injection (0 h to 24 h), the animals were treated with ethyl ether to induce light amnesia. The abdominal cavity was opened, blood was collected into a heparinized centrifuge tube through a catheter inserted into the abdominal aorta to measure biochemical values, and the kidney was then perfused in situ with physiological saline until the venous effluent became clear and then removed. Kidneys were immediately frozen in liquid nitrogen and stored at −80°C. For histology, kidney tissue was fixed in 10% neutral buffered formalin, embedded in paraffin, sectioned to 4–6 μm, and stained with hematoxylin and eosin.

### Preparation of cDNA probes

The template cDNAs used to generate probes for Northern blot analysis were rat pRHO-1 [8], provided by Dr. S Shibahara (Tohoku University, Sendai, Japan), rat pKRA2cA [9], ALAS1, provided by Dr. M Yamamoto (Tohoku University, Sendai, Japan), and rat Bach1 corresponding to base pairs 785−1382, provided by Dr. K Igarashi (Tohoku University, Sendai, Japan). The rat Bach1 cDNA was prepared from C6 glioma RNA using reverse-transcription and the polymerase chain-reaction and inserted into pCR-BluntII-TOPO (Invitrogen) [10]. All cDNA probes used for northern blot analysis were labeled with [α-^32^P] dCTP (PerkinElmer, Inc., Japan) using the High Prime DNA Labeling Kit (Roche, Basel, Swiss) according to the manufacturer’s instructions.

### RNA isolation and Northern blot analysis

Total RNA was isolated from the rat tissues using TRI-Reagent (Molecular Research Center, Inc. Cincinnati, USA) according to the manufacturer’s protocol. Northern blot analysis was performed as described previously [11]. Briefly, total RNA (20 μg) was separated by electrophoresis through a 1.2% (w/v) agarose gel containing 6.5% (v/v) formaldehyde. After blotting on a sheet of Bio-Rad Zeta-Probe GT Blotting Membrane (Bio-Rad Laboratories, Richmond, CA, USA), RNA samples were hybridized with [α-^32^P] dCTP-labeled cDNA probes, followed by washing under stringent conditions. Each blotted membrane was placed on a sheet of Fuji Medical X-ray film (Fujifilm Co., Tokyo, Japan) with an intensifying screen at −80°C. Target and 18S ribosomal RNA bands were visualized and quantified using an image scanner (ChemiDoc XRS Plus Imaging System, Bio-Rad, USA) and image analysis software (Image Lab Version 5.0, Bio-Rad). The relative amounts of hybridized radiolabeled cDNAs were normalized to 18S ribosomal RNA levels to correct for differences in sample loading.

### Preparation of cell extracts, microsomal and nuclear-cytosolic fractionation

Kidneys were homogenized in three volumes of 1.15% (w/v) KCl and centrifuged at 10,000*g* for 10 min at 4°C, followed by centrifugation of the supernatant at 105,000*g* for 60 min at 4°C to obtain the microsomal fraction. The pellet was resuspended in 0.1 M potassium phosphate buffer (pH 7.4) containing 10% glycerol. The nuclear and cytoplasmic proteins were extracted using NE-PER Nuclear and Cytoplasmic Extraction Reagents (Pierce, Rockford, IL, USA) according to the manufacturer’s instructions. Cytoplasmic proteins from kidney homogenates were extracted with ice-cold Cytoplasmic Extraction Reagent 1 (CER1) containing a protease inhibitor followed by CER2. After CER1 and CER2 were added to the sample, the mixture was centrifuged at 16,000*g* 5 min at 4°C to obtain the cytoplasmic fraction as the supernatant. Next, the nuclear proteins were extracted from the pellet with ice-cold NER containing a protease followed by centrifugation of the supernatant for 10 min at 4°C. Proteins concentrations were determined using the BCA assay (Pierce, Rockford, IL, USA).

### Western blot analysis

Western blotting was performed as described previously [12]. Samples equivalent to 25 μg of protein were applied to 7.5% or 12.5% (w/v) polyacrylamide-SDS gels. After electrophoretic separation, the proteins were transferred to Amercham Hybond-polyvinylidene fluoride (PVDF) membranes (GE Healthcare Japan Co., Tokyo, Japan). The membrane was blocked with 4% (w/v) BLOCK ACE solution for 1 h at room temperature, and incubated overnight at 4°C with each primary antibody diluted with Tris-buffered saline containing Tween 20 (rabbit anti-HO-1 polyclonal antibody 1:1000 dilution, SPA-896, Assay Designs; rabbit anti-Bach1 polyclonal antibody 1:1000 dilution, #14018-1-AP, Proteintech; mouse anti-β-actin monoclonal antibody 1:5000 dilution, sc-47778, Santa Cruz; goat anti-lamin B polyclonal antibody 1:5000 dilution, sc-6216, Santa Cruz; and mouse monoclonal antibody to α-tubulin 1:5000 dilution, sc-8035). The loading control was lamin B for nuclear protein and β-actin for cytosolic and microsomal protein. After washing with buffer, the membrane was treated with a 1:10000 dilution of horseradish peroxidase-labeled secondary antibody for 30 min at room temperature (goat anti-rabbit IgG-HRP, sc-2004, Santa Cruz for HO-1 and Bach1; goat anti-mouse IgG-HRP, sc-2005, Santa Cruz, for β-actin; rabbit anti-goat IgG-HRP, sc-2768, Santa Cruz, for lamin B; or goat anti-mouse IgM-HRP, sc-2064, Santa Cruz, for α-tubulin). The membranes were stained with Clarity Western ECL Substrate (Bio-Rad, USA). Antigen–antibody complexes were visualized using an image scanner (ChemiDoc XRS Plus Imaging System, Bio-Rad) and the signals were quantified using analysis software (Image Lab Version 5.0, Bio-Rad).

### Biochemical assays

Assays of serum blood urea nitrogen (BUN), creatinine (Cr), creatinine phosphokinase (CK), and aspartate aminotransferase (AST) activity were performed to evaluate AKI and rhabdomyolysis. Serum was separated from whole blood by centrifugation at 1600*g* for 10 min at RT. The serum was decanted and stored at −80°C, and BUN, Cr, CK, and AST were measured using an automated biochemical analyzer (Fuji DRI-CHEM 7000i^;^ Fujifilm Co., Tokyo, Japan).

### Histological analysis

The kidney was isolated immediately after killing the animal and washed with ice-cold saline. For histological examinations, tissues were fixed in 10% neutral buffered formalin, embedded in paraffin, and sectioned at thicknesses of 4−6 μm. After deparaffinization and dehydration, the sections were stained with hematoxylin and eosin (HE) and examined by a nephrologist using a light microscope. Images of representative fields were recorded. Blinded scoring analysis was performed. Tubular injury was defined as tubular epithelial cell swelling, vacuolar degeneration, necrosis, and desquamation. Tubular injury score was graded by estimating the percentage of injured tubules as follows: 0, areas of injured tubules < 5%; 1, areas of injured tubules 5%–25%; 2, areas of injured tubules 26%–50%; 3, areas of injured tubules 51%–75%; and 4, areas of injured tubules > 75%, as reported previously [13]. All evaluations were performed on 10 random fields for each kidney and 5 kidneys for each group. Results are expressed as the mean score of tubular injury scores per group.

### Statistical analysis

Data are presented as the mean ± standard deviation. For statistical evaluation, two-way ANOVA without replication followed by Tukey’s honestly significant difference test or the unpaired Student *t* test was used. JMP10 software (SAS Institute Inc., Cary, NC, USA) was used for all statistical calculations. P < 0.05 indicated a statistically significant difference.

## Results

### Effect of glycerol treatment on serum CK and AST levels

**Fig 1:**
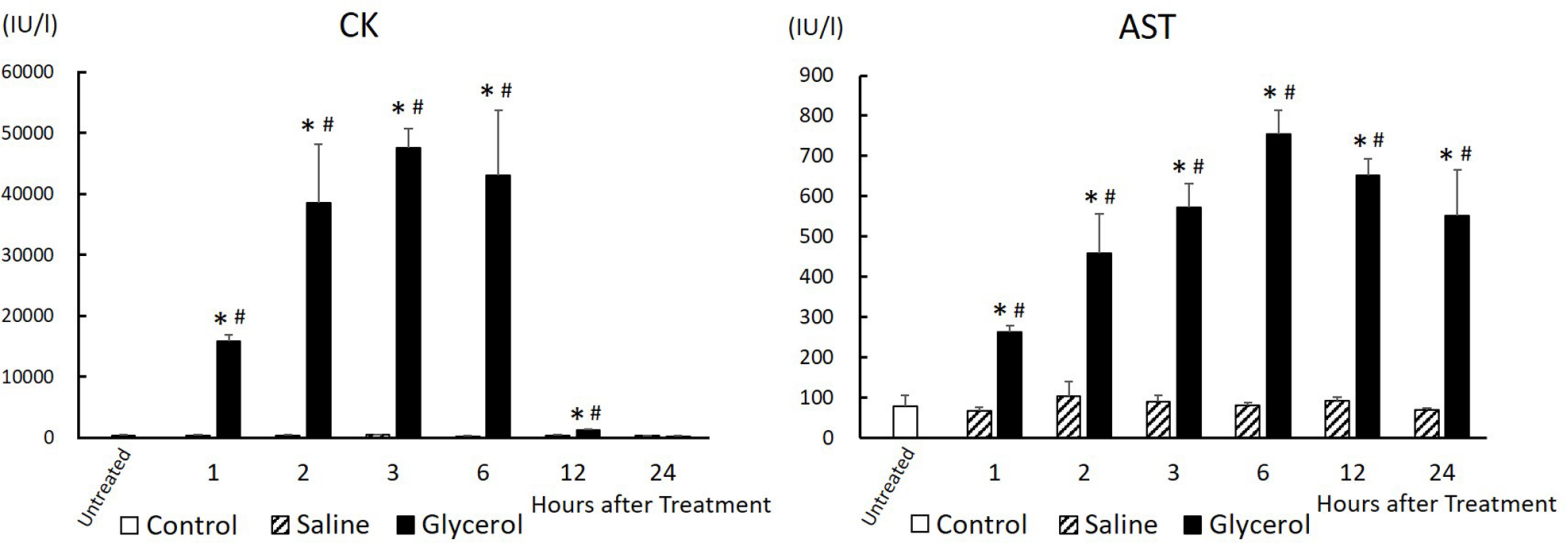
Changes in serum CK and AST levels after glycerol treatment. Rats treated with 50% glycerol (10 mL/kg, i.m.) or saline (10 mL/kg, i.m.) were sacrificed 1, 2, 3, 6, 12, and 24 h after injection. Serum CK and AST levels were measured at each time. Control, untreated control rats; Saline; saline-treated control rats; Glycerol, glycerol-treated rats. Data are expressed as the mean ± standard error (n = 3–6). Multiple comparisons were analyzed using two-way ANOVA without replication followed by Tukey's honestly significant difference test. *significantly different from untreated control rats (P < 0.05). #significantly different from rats receiving saline at the corresponding time (P < 0.05). CK, creatinine phosphokinase. AST, aspartate aminotransferase.

We first administered 10 mL/kg of 50% glycerol to rats and examined its effect on the serum levels of CK and AST (Fig 1). Following glycerol treatment, the serum CK levels rapidly increased within 1 h, increased further, and reached a maximal level at 3 h, maintained until 6 h, rapidly declined, and returned to approximately basal levels at 12 h, while the levels of the saline-treated group were normal throughout the experiment. Similar to the changes in the serum CK levels, the serum AST levels in the glycerol-treated group started to increase at 1 h after treatment, increased linearly, peaked at 6 h, and increased continuously until 24 h. In contrast, saline treatment did not influence serum AST levels at any time. These results indicate that the glycerol treatment caused severe rhabdomyolysis in rats.

### Effect of glycerol treatment on serum BUN and Cr levels

**Fig 2:**
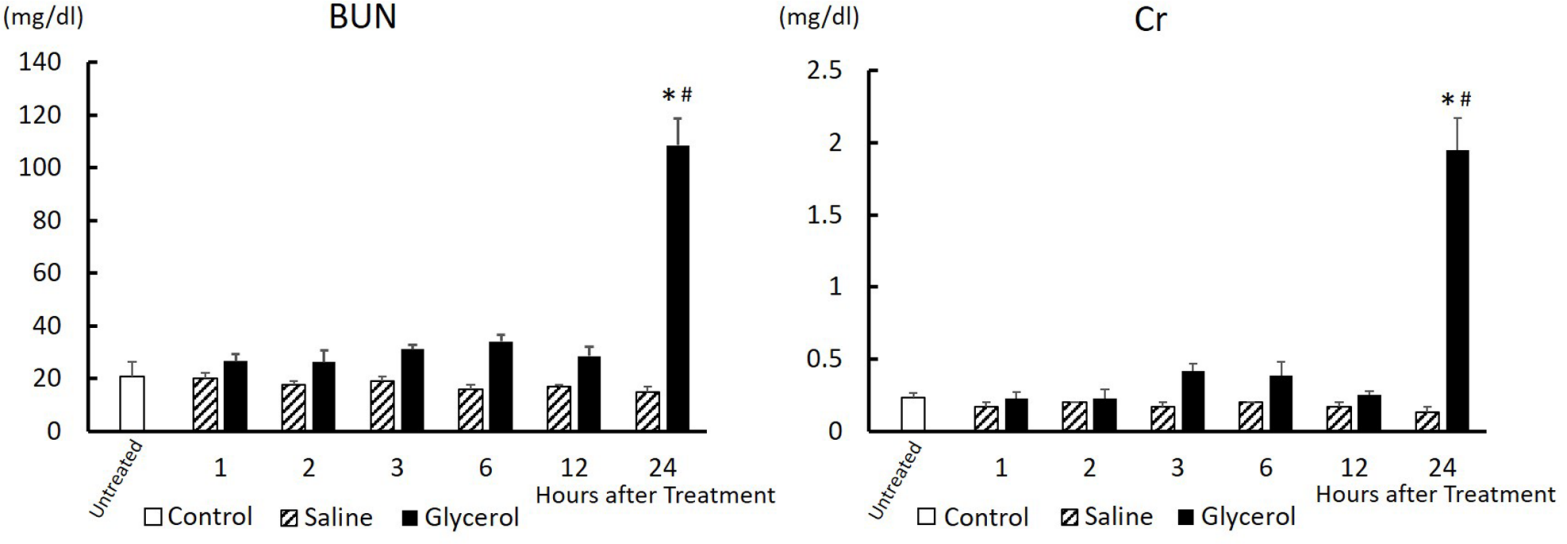
Changes in serum BUN and Cr levels after glycerol treatment. Rats treated with 50% glycerol (10 mL/kg, i.m.) or saline (10 mL/kg, i.m.) were sacrificed at 1, 2, 3, 6, 12, and 24 h after injection. Serum BUN and Cr levels were measured at each time after treatment. Control, untreated control rats; Saline, saline-treated control rats; Glycerol, glycerol-treated rats. Data are expressed as the mean ± standard error (n = 3–6). Multiple comparisons were analyzed using two-way ANOVA without replication followed by Tukey's honestly significant difference test. *significantly different from untreated control rats (P < 0.0001). #significantly different from rats receiving saline 24 h after treatment (P < 0.0001). BUN, blood urea nitrogen. Cr, creatinine.

**Fig 3:**
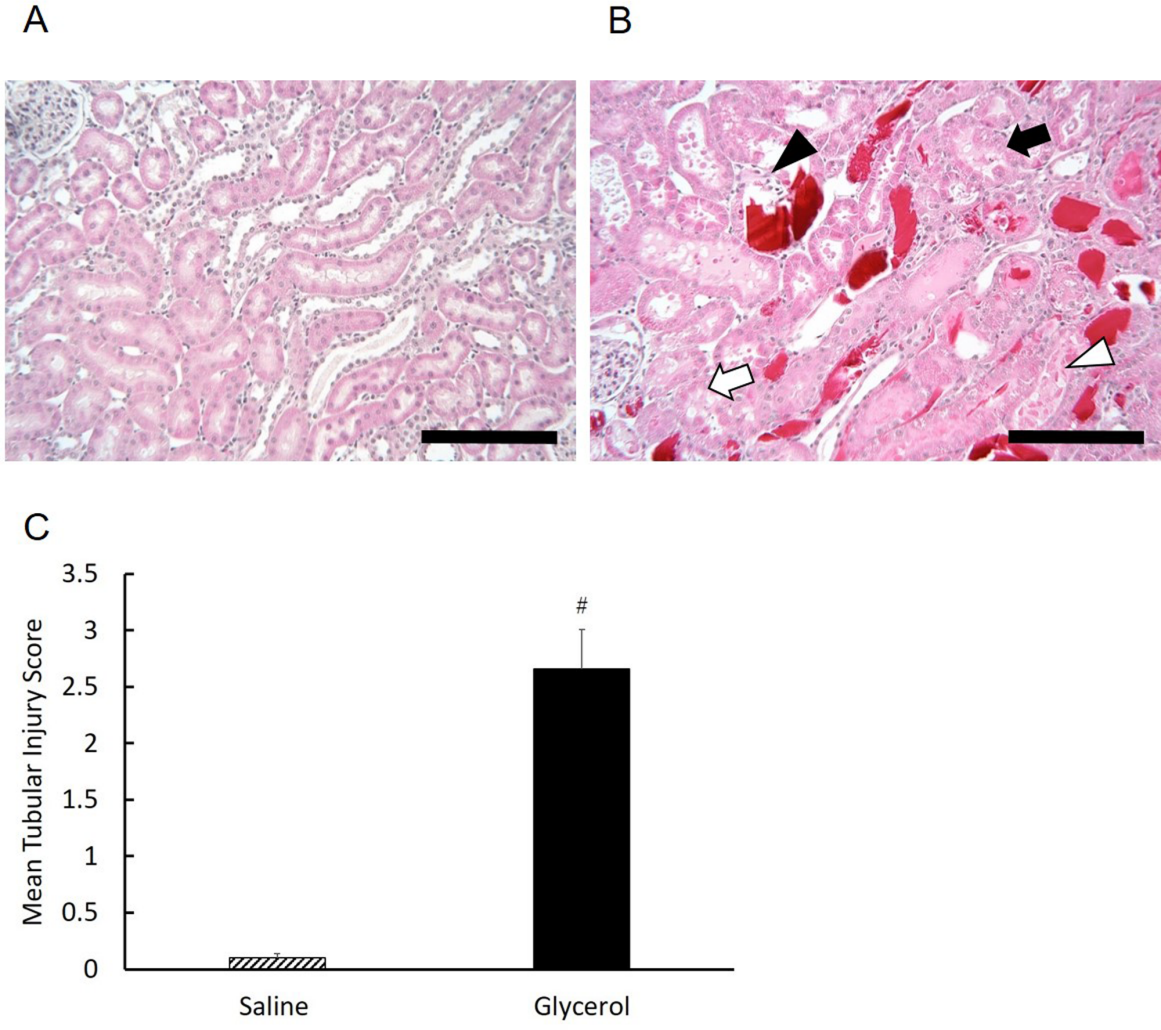
Renal histopathological changes after glycerol treatment. Renal tissues were stained with HE and examined using a light microscope. Representative images 24 h after treatment (HE staining, original magnification ×200, scale bar = 100 μm). A. Saline group, normal healthy histology. B. Glycerol group, widespread tissue damage. Black arrow, open arrow, black arrowhead, and open arrowhead indicate tubular epithelial swelling, vacuolar degeneration, necrosis, and desquamation, respectively. C. The severity of histopathological changes. A total of 50 areas from a renal section from each group were evaluated. Tubular injury was defined as tubular epithelial cell swelling, vacuolar degeneration, necrosis, and desquamation. Tubular injury score was graded by estimating the percentage of injured tubules as follows: 0, areas of injured tubules < 5%; 1, areas of injured tubules 5%–25%; 2, areas of injured tubules 26%–50%; 3, areas of injured tubules 51%–75%; and 4, areas of injured tubules > 75%. The mean score for each kidney is provided, followed by the mean score for the group. Data are expressed as the mean ± standard error. For statistical evaluation, the unpaired Student *t* test was used. #significantly different from rats receiving saline (P < 0.0001).

Next, we examined the change in serum BUN and Cr levels as measures of renal function as well as the histological changes of the kidney after treatment. BUN and Cr levels in glycerol-treated rats were not significantly different compared those of the saline-treated rats for the first 12 h (Fig 2). However, 24 h after treatment, both indices in the glycerol-treated rats were markedly increased, and the levels were significantly higher compared with those of the saline-treated group (BUN, 108.45 ± 20.59 md/dl vs 14.97 ± 3.44 mg/dl, P < 0.0001; Cr, 1.95 ± 0.44 mg/dl vs 0.13 ± 0.06 mg/dl, P < 0.0001). The levels of BUN and Cr of the glycerol-treated group were approximately 7-fold and 15-fold higher compared with those of the saline group, respectively (Fig 2). Histological examination of kidneys at 24 h after glycerol treatment revealed injuries to tubule epithelial cells, predominantly affecting in cortex, with cast formation in the medulla (Fig 3B). In saline-treated animals, renal injury was not observed (Fig 3A). The significant effect of glycerol was also confirmed by the scoring of tubular injury by independent researchers blinded to the treatment. Glycerol-induced tubular injury was confirmed by an increase in the mean tubular injury score (Fig 3C). These results indicate that the glycerol treatment caused rhabdomyolysis-associated acute kidney injury in rats.

### Effect of glycerol treatment on renal HO-1 expression in the kidney

**Fig 4:**
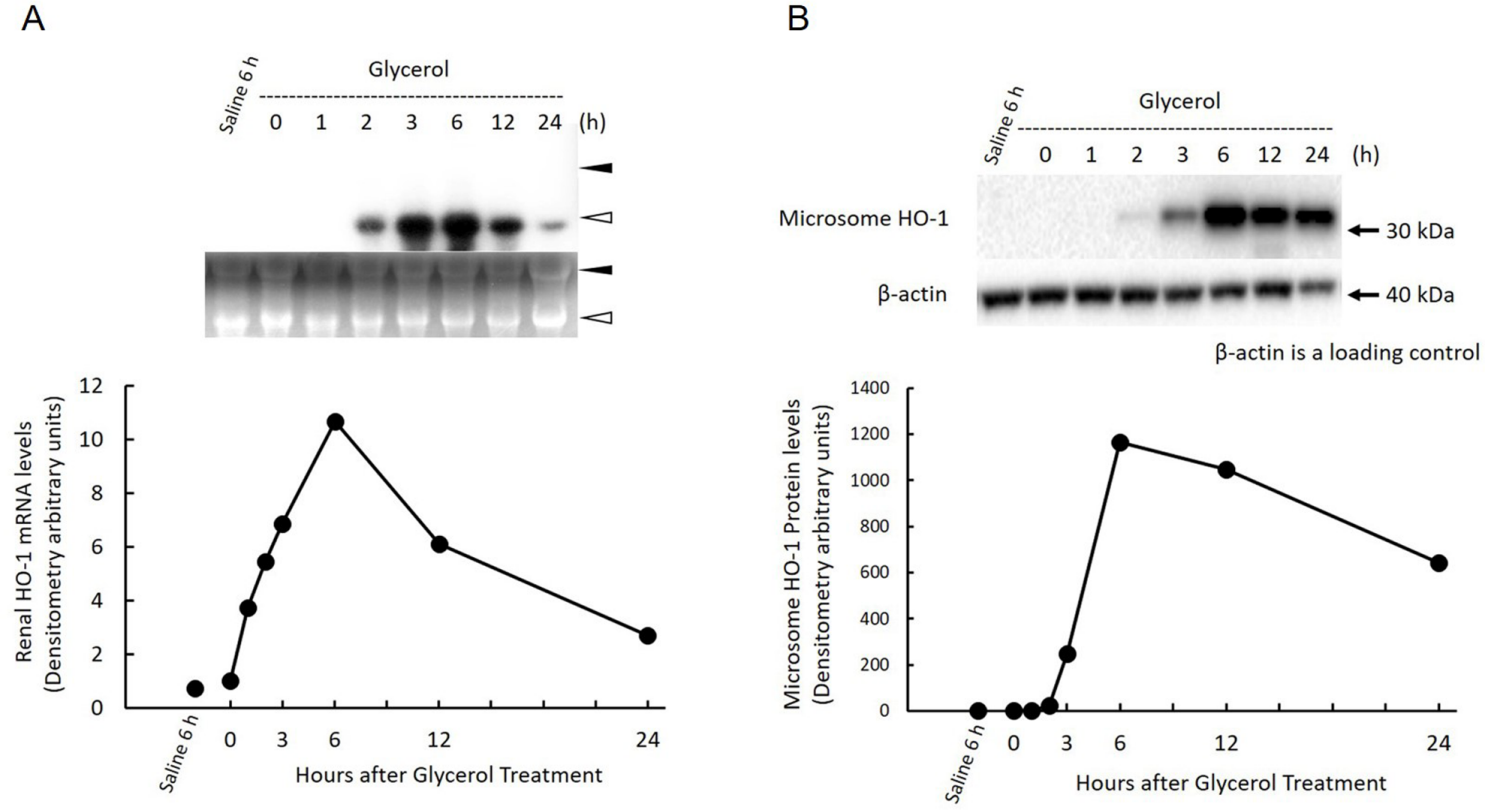
Changes in HO-1 gene and protein expression in the kidney after glycerol treatment. Rats treated with 50% glycerol (10 mL/kg, i.m.) were sacrificed 0, 1, 2, 3, 6, 12, and 24 h after injection, and kidneys were removed for northern and western blot analysis as described in Materials and Methods. A) Top panel: 20 μg of total RNA was subjected to northern blot analysis. Autoradiographic signals of RNA blots hybridized with a [α-^32^P] dCTP-labeled HO-1 cDNA probe. Three independent experiments yielded similar results, and a typical example is shown. Ethidium bromide staining of the same RNA is shown as a loading control. Closed arrowhead, 28S ribosomal RNA; open arrowhead, 18S ribosomal RNA. Bottom panel: Time-course changes in renal HO-1 gene expression after glycerol treatment. HO-1 gene expression levels are expressed as arbitrary densitometric units. B) Top panel: samples equivalent to 25 μg of protein were subjected to western blot analysis. Shown are the chemiluminescent signals of protein blots reacted with a rabbit polyclonal anti-rat HO-1 antibody. Three independent experiments yielded similar results, and a typical example is shown. Bottom panel: Time-course changes in renal HO-1 protein expression after glycerol treatment. The concentrations of renal HO-1 protein are expressed as densitometric arbitrary units.

We next examined the time course changes in gene and protein expression of HO-1in the kidney. HO-1 mRNA was barely detectable in the saline-treated control kidney. In contrast, after glycerol treatment, HO-1 mRNA levels started to increase linearly at 1 h, reaching a maximum at 6 h, decreasing to 50% of the maximum level at 12 h, and gradually decreasing to 20% of the maximal level at 24 h (Fig 4A). Consistent with the changes in HO-1 mRNA, HO-1 protein expression was significantly increased at 3 h after glycerol treatment, abruptly increased, reached a maximum level at 6 h, and gradually decreased to approximately 50% of the maximum level at 24 h (Fig 4B). HO-1 protein was not detectable in the saline-treated control kidney.

### Effect of glycerol treatment on renal ALAS1 gene expression in the kidney

**Fig 5:**
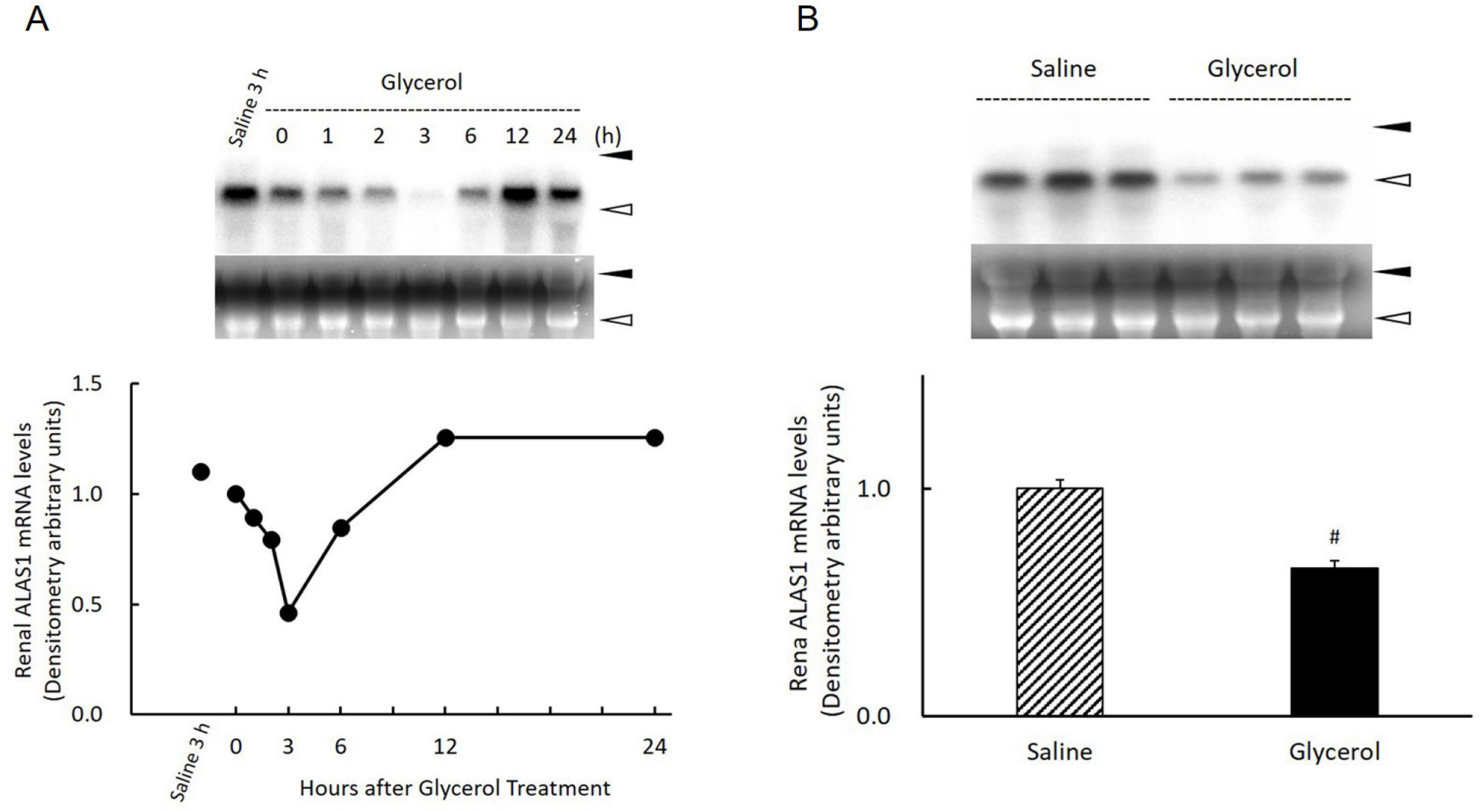
Changes in ALAS 1 gene expression in the kidney after glycerol treatment. Rats treated with 50% glycerol (10 mL/kg, i.m.) were sacrificed 0, 1, 2, 3, 6, 12, and 24 h after injection and kidneys were removed for northern blot analysis as described in Materials and Methods. A) Top panel: 20 μg of total RNA was subjected to northern blot analysis. Autoradiographic signals of RNA blots hybridized with an [α-^32^P] dCTP-labeled ALAS1 cDNA probe. Three independent experiments yielded similar results, and a typical example is shown. Ethidium bromide staining of the same RNA is shown as a loading control. Closed arrowhead, 28S ribosomal RNA; open arrowhead, 18S ribosomal RNA. Bottom panel: Time-course changes in renal ALAS1 gene expression after glycerol treatment. ALAS1 gene expression levels are expressed as arbitrary densitometric units. B) Top panel: 20 μg of total RNA was subjected to northern blot analysis. Autoradiographic signals of RNA blots hybridized with an [α-^32^P]dCTP-labeled ALAS-1 cDNA probe. Ethidium bromide staining of the same RNA is shown as a loading control. Closed arrowhead, 28S ribosomal RNA; open arrowhead, 18S ribosomal RNA. Bottom panel: Comparison of renal ALAS1 gene expression 3 h after saline treatment or glycerol treatment (n = 3). ALAS1 gene expression levels are expressed as arbitrary densitometric units. Data are expressed as the mean ± standard error. For statistical evaluation, the unpaired Student *t* test was used. #significantly different from rats receiving saline at 3 h (P = 0.0021).

HO-1 is induced by heme, and the expression of ALAS1, the rate-limiting enzyme in heme biosynthesis, is down-regulated by heme. Therefore, we determined the time course changes of ALAS1 gene expression in the kidney after glycerol treatment. ALAS1 mRNA was significantly expressed in the saline-treated control kidney at 3 h. In contrast to HO-1 gene expression, following glycerol treatment, the renal ALAS1 mRNA level decreased rapidly at 1 h, reached a minimum at 3 h, gradually increased to greater than basal levels at 12 h, and the plateaued by 24 h (Fig 5A). When the levels of renal ALAS1 mRNA in glycerol treated-animals were compared with those from saline-treated animals at 3 h, the average level of the glycerol-treated group was significantly lower compared with that of the saline-treated group (Fig 5B).

### Effect of glycerol treatment on renal Bach1 protein expression in the nucleus and cytosol

**Fig 6:**
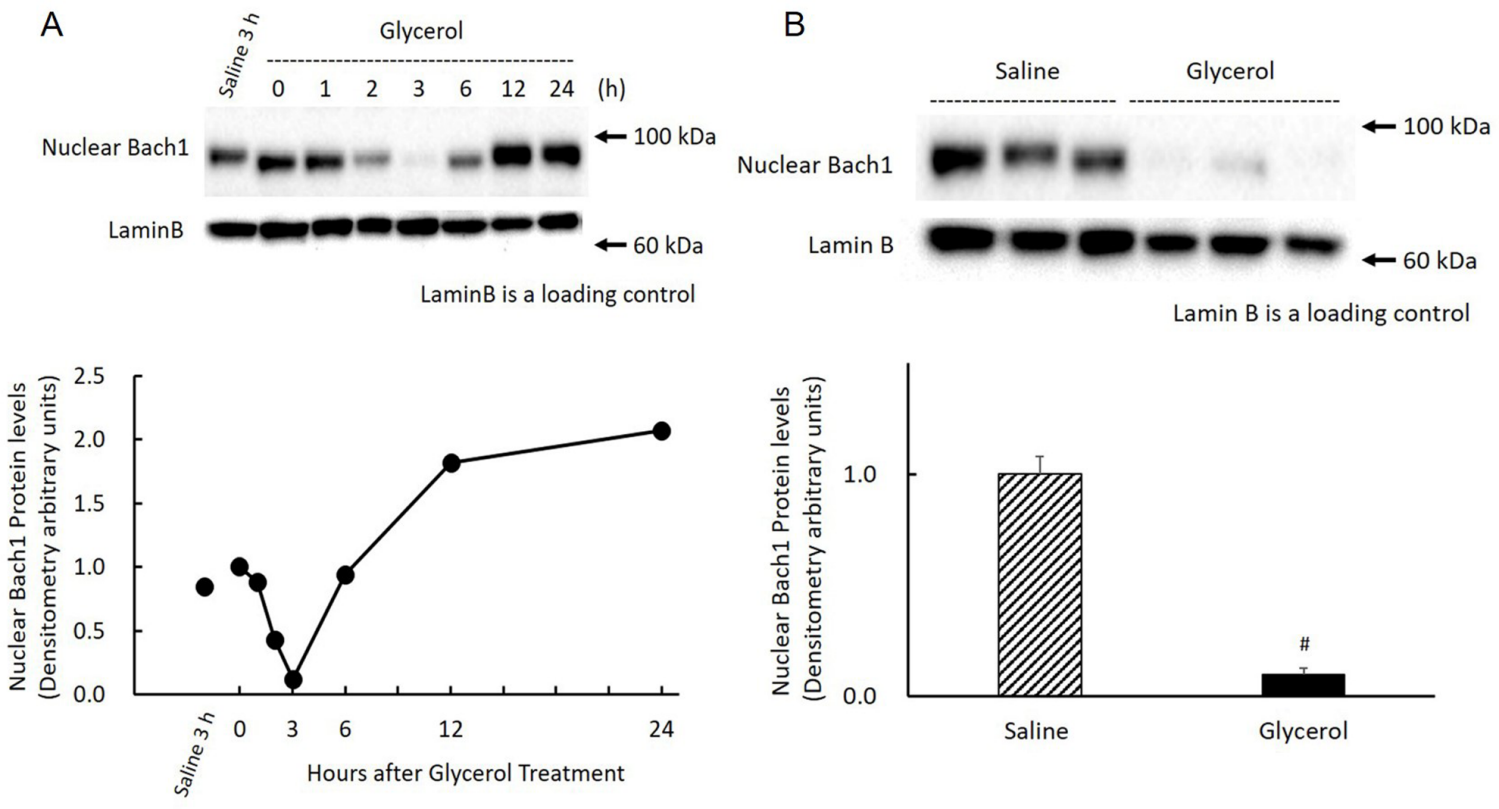
Changes in renal nuclear Bach1 protein after glycerol treatment. Rats treated with 50% glycerol (10 ml/kg, i.m.) were sacrificed 0, 1, 2, 3, 6, 12, and 24 h after the injection and kidneys were removed for western blot analysis as described in Materials and Methods. A) Top panel: samples equivalent to 25 μg of protein were subjected to western blot analysis. Shown are the chemiluminescent signals of protein blots reacted with a rabbit polyclonal anti-rat Bach1 antibody. Three independent experiments yielded similar results, and a typical example is shown. Bottom panel: Time-course changes in renal nuclear Bach1 protein expression after glycerol treatment. The concentrations of nuclear Bach1 protein are expressed as arbitrary densitometric units. B) Top panel: samples equivalent to 25 μg of protein were subjected to western blot analysis. Shown are the chemiluminescent signals of protein blots reacted with a rabbit polyclonal anti-rat Bach 1 antibody. Three independent experiments yielded similar results, and a typical example is shown. Bottom panel: Comparison of renal nuclear Bach1 protein expression 3 h after saline treatment or glycerol treatment (n = 3). Nuclear Bach1 protein expression is expressed as arbitrary densitometric units. Data are expressed as the mean ± standard error. For statistical evaluation, the unpaired Student *t* test was used. #significantly different from rats receiving saline at 3 h (P < 0.0001).

**Fig 7:**
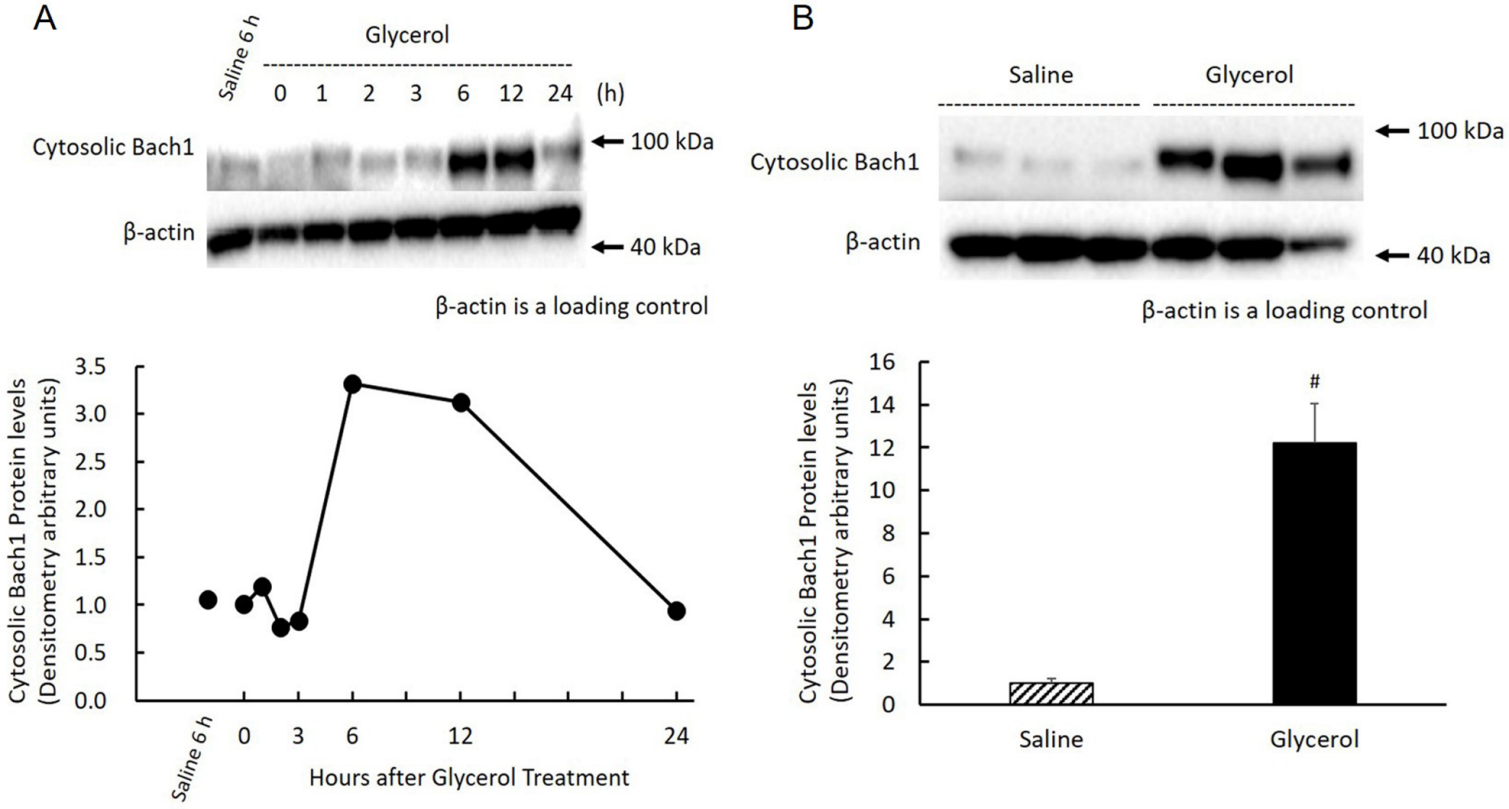
Changes in renal cytosolic Bach1 protein after glycerol treatment. Rats treated with 50% glycerol (10 mL/kg, i.m.) were sacrificed 0, 1, 2, 3, 6, 12, and 24 h after the injection, and kidneys were removed for western blot analysis as described in Materials and Methods. A) Top panel: samples equivalent to 25 μg of protein were subjected to western blot analysis. Shown are the chemiluminescent signals of protein blots reacted with a rabbit polyclonal anti-rat Bach1 antibody. Three independent experiments showed similar results, and a typical example is shown. Bottom panel: Time-course changes in renal cytosolic Bach1 protein expression after glycerol treatment. The concentrations of cytosolic Bach1 protein are expressed as arbitrary densitometric units. B) Top panel: samples equivalent to 25 μg of protein were subjected to western blot analysis. Shown are the chemiluminescent signals of protein blots reacted with a rabbit polyclonal anti-rat Bach 1 antibody. Three independent experiments yielded similar results, and a typical example is shown. Bottom panel: Comparison of renal cytosolic Bach1 protein expression 6 h after saline treatment or glycerol treatment (n = 3). Cytosolic Bach1 protein expression is expressed as arbitrary densitometric units. Data are expressed as the mean ± standard error. For statistical evaluation, the unpaired Student *t* test was used. #significantly different from rats receiving saline at 6 h (P = 0.0037).

There was a reciprocal relationship between HO-1 and ALAS1 expression, suggesting an increase in free heme concentration in the glycerol-treated kidney. Bach1 is a heme-responsive transcription repressor that regulates the expression of the HO-1 gene. We therefore examined the time course changes of Bach1 protein expression in the nucleus as well as in the cytosol of glycerol-treated kidney cells. Nuclear Bach1 protein in the kidney was significantly expressed in the saline-treated control kidney. Following glycerol treatment, renal nuclear Bach1 protein levels rapidly and significantly decreased and reached a nadir at 3 h, followed by a gradual restoration, which exceeded the normal level at 12 h, and maintained at high level by 24 h (Fig 6A). When we determined nuclear Bach1 protein levels in glycerol treated-animals with those from saline-treated animals 3 h after saline-treatment, the average level of the glycerol-treated group was <10% of the control group (P < 0.0001) (Fig 6B). In contrast to the expression of nuclear Bach1 protein, the levels of cytosolic Bach1 protein levels abruptly increased and reached a maximum 6 h after treatment, which were maintained up to 12 h, gradually decreased, and returned to basal levels at 24 h. However, the levels for the first 3 h after treatment was not significantly different compared with those of the saline-treated kidney (Fig 7A). Compared with cytosolic Bach1 protein levels at 6 h in glycerol-treated animals with those in saline-treated animals 6 h after saline-treatment, the average level of the glycerol-treated group was >10-times higher compared with that of the saline-treated group (P = 0.0037) (Fig 7B).

### Effect of glycerol treatment on Bach1 gene expression in the kidney

**Fig 8:**
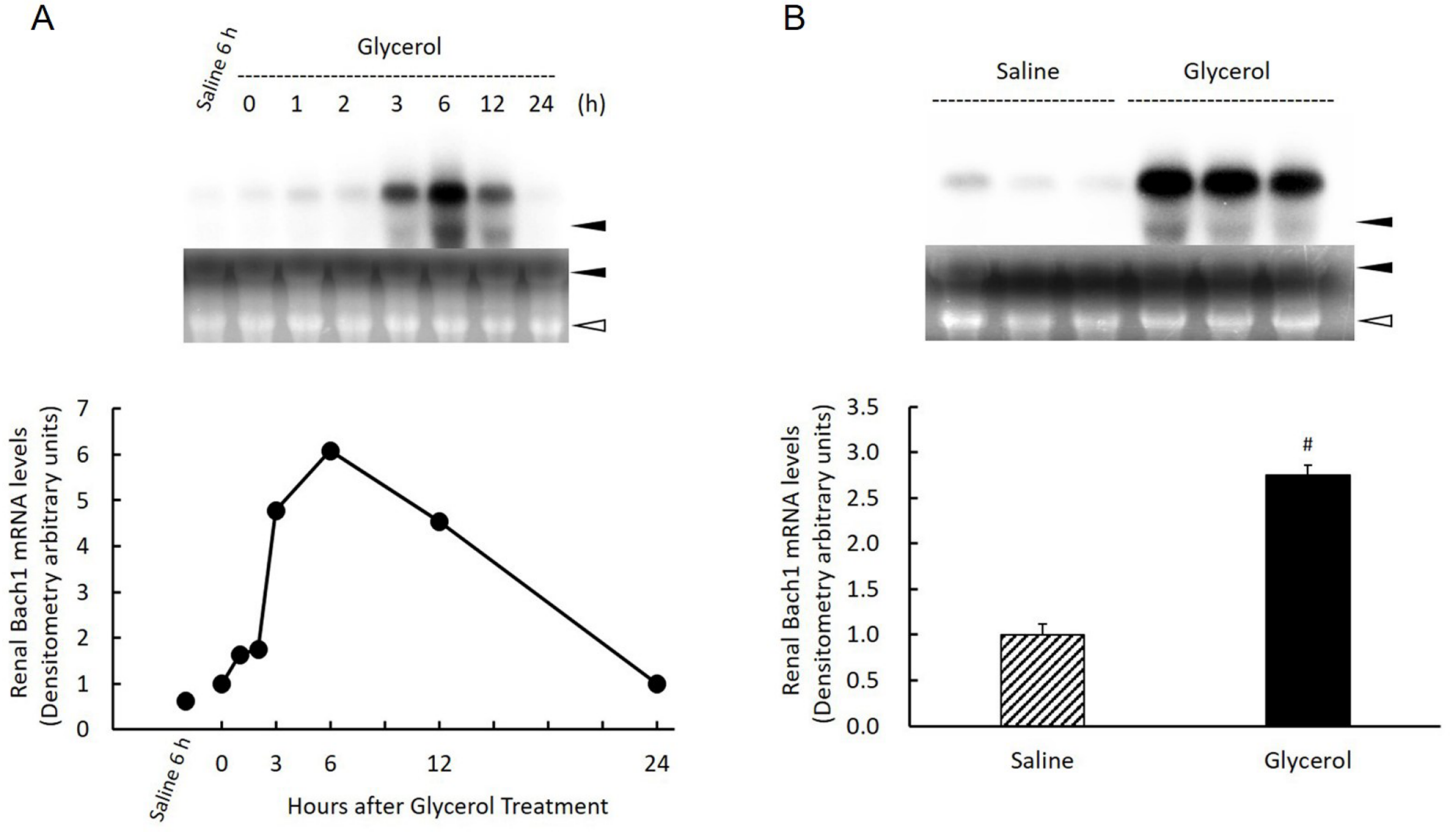
Changes in renal Bach1 gene expression after glycerol treatment. Rats treated with 50% glycerol (10 mL/kg, i.m.) were sacrificed 0, 1, 2, 3, 6, 12, and 24 h after the injection and kidneys were removed for northern blot analysis as described in Materials and Methods. A) Top panel: 20 μg of total RNA was subjected to northern blot analysis. Autoradiographic signals of RNA blots hybridized with an [α-^32^P]dCTP-labeled Bach1 cDNA probe. Three independent experiments yielded similar results, and a typical example is shown. Ethidium bromide staining of the same RNA is shown as a loading control. Closed arrowhead, 28S ribosomal RNA; open arrowhead, 18S ribosomal RNA. Bottom panel: Time-course changes of renal Bach1 gene expression after glycerol treatment. Bach1 gene expression levels expressed as arbitrary densitometric units. B) Top panel: 20 μg of total RNA was subjected to northern blot analysis. Autoradiographic signals of RNA blots hybridized with an [α-^32^P]dCTP-labeled Bach1 cDNA probe. Ethidium bromide staining of the same RNA is shown as a loading control. Closed arrowhead, 28S ribosomal RNA; open arrowhead, 18S ribosomal RNA. Bottom panel: Comparison of renal Bach1 gene expression 6 h after saline treatment or glycerol treatment (n = 3). Bach1 gene expression levels are expressed as arbitrary densitometric units. Data are expressed as the mean ± standard error. For statistical evaluation, the unpaired Student *t* test was used. #significantly different from rats receiving saline at 6 h (P = 0.0004).

Glycerol treatment induced significant changes in protein expression in the nucleus and cytosol in cells of the kidney, suggesting that it may influence *de novo* synthesis of Bach1 as well as changing its intracellular distribution. Therefore, we examined the effect of glycerol treatment on Bach1 gene expression in the kidney. While Bach1 mRNA was hardly detectable in the saline-treated control kidney, it increased significantly 3 h after glycerol treatment, reached a maximum at 6 h, which was maintained up to 12 h, decreased gradually, and then returned to approximately basal levels after 24 h. When the levels of renal Bach1 mRNA in glycerol-treated animals were compared with those from saline-treated animals 6 h after saline-treatment, the average level of the glycerol-treated group was approximately 2.5-times higher compared with that of the saline-treated group (P = 0.0004) (Fig 8B).

## Discussion

In the present study, we demonstrate that nuclear Bach1 protein was rapidly and significantly decreased in the kidneys of rats with glycerol-associated RM-AKI, followed by an increase in Bach1 protein in the cytosol, which was preceded by the induction of Bach1 mRNA. We detected a significant increase in HO-1 expression and the robust inhibition of ALAS1 expression in the kidneys of glycerol-treated animals, suggesting a significant increase in the free heme concentration in the kidney of glycerol-treated animals. Although we confirmed the entire time-course change of each parameter in the saline group in advance, saline treatment did not influence each expression throughout the experimental period. Bach1 is a heme-responsive transcription repressor of the HO-1 gene, and our findings suggest that changes in the subcellular distribution of Bach1 may be involved in the induction of HO-1 accompanying heme metabolism in the kidney of the rat RM-AKI model. To the best of our knowledge, this is the first study to show dynamic changes in renal Bach1 expression *in vivo*, which were associated with heme metabolism.

We show further that HO-1 mRNA and protein levels significantly increased, while gene expression of ALAS1, the rate limiting enzyme in heme biosynthesis, was significantly inhibited after glycerol treatment. HO-1 is significantly increased in the kidneys of glycerol-treated animals as an adaptive response to RM-AKI [2, 3]. The inverse kinetics of HO-1 and ALAS1 gene expression observed in this study strongly suggest that there may be a significant increase in the renal intracellular free-heme concentration after glycerol treatment, because HO-1 is up-regulated [14–16] while ALAS1 is down-regulated by free heme [9, 17, 18]. Consistent with this hypothesis, our previous report shows that there is a rapid increase in microsomal heme concentrations following reperfusion in a rat model of ischemic acute renal failure, another acute kidney injury, which is accompanied by the induction of renal HO-1 and the inhibition of renal ALAS1, reflecting an increase in free heme concentration in the kidney [19].

In this study, we have demonstrated a dramatic increase only in the expression of HO-1 mRNA and protein. Our previous reports demonstrated a marked increase in HO activity following HO-1 gene and protein induction in several rat organ injury models [19–21]. In particular, Shimizu et al. demonstrated a significant increase in HO activity following an increase in the accumulation of HO-1 mRNA and protein in renal microsomal heme in rat ischemic acute renal failure models [19]. Based on these findings, we suggested that an increase in the levels of renal HO-1 mRNA and protein in rat RM-AKI models might also accompany HO activation [2].

“Free heme” represents newly synthesized heme not complexed with its apohemoprotein, or free heme released from a hemo protein but not yet cleaved by HO. Although heme is required as the prosthetic group for hemoproteins that are crucial for cellular viability, an excess amount of free heme can be deleterious, because it can intercalate into the phospholipid bimolecular membrane to generate oxygen free radicals as a lipophilic iron, ultimately leading to cellar injury [22]. Although the nature of increased free heme concentrations in the kidney after glycerol treatment is unclear, it is likely that heme released from degraded myoglobin may be the source of “free heme” that can induce RM-AKI. Consistent with our hypothesis, a significant amount of myoglobin released from damaged muscle cells is reabsorbed into the proximal tubule cells after its glomerular filtration in rhabdomyolysis [23].

Bach1 acts as a negative regulator of the transcription of HO-1 *in vitro*. In cultured cells, under normal conditions, the Bach1-small Maf heterodimer binds to Maf the recognition element (MARE) and represses HO-1 gene expression [24]. An increase in the intracellular heme concentration can release Bach1 from MARE and promote Bach1 nuclear export by directly binding to heme, which allows transcriptional activation of HO-1 [4–7, 25]. Moreover, overexpression of Bach1 represses the induction HO-1 expression by heme in cultured cells in response to exposure to ultraviolet-A light [26]. Thus, the modulation of Bach1 is considered indispensable for the induction of HO-1 by heme.

After glycerol treatment, renal nuclear Bach1 protein levels rapidly and significantly decreased, reaching a nadir after 3 h. In response to the induction of HO-1 mRNA, protein synthesis rapidly ensued, reaching a maximum after 6 h. Thus, the reduction of nuclear Bach1 protein preceded the increase in HO-1 expression in the kidneys of glycerol-treated animals, suggesting that nuclear Bach1 protein was displaced from MARE and exported from the nucleus by directly binding to heme released from myoglobin, ultimately leading to HO-1 induction. Similar to the experiments using cultured cells, our findings strongly suggest that intranuclear Bach1 inhibits the transcription of HO-1 in normal rat kidney and that the deprivation of nuclear Bach1 led to renal HO-1 induction in RM-AKI. To date, no *in vivo* reports have demonstrated the nuclear export of Bach1 and induction of HO-1, which is probably related to the increase in intracellular heme.

In contrast to nuclear Bach1 protein expression, cytosolic Bach1 protein levels significantly increased, reaching a maximum 6 h after the treatment. The time to attain the maximum level of cytosolic protein expression was delayed by 3 h relative to the minimum level of nuclear Bach1 protein expression. An increase in cytosolic Bach1 protein was followed by a decrease in nuclear Bach1 protein. These changes did not occur at the same time; there was a significant discrepancy between the time points for the increase of cytosolic Bach1 protein and the decrease of nuclear Bach1 protein. Our results also revealed that the Bach1 mRNA levels significantly and rapidly increased 3 h after glycerol treatment. The elevated expression of Bach1 mRNA was concurrent with a significant decrease in nuclear Bach1 protein and was preceded by a robust increase in cytosolic Bach1 protein. It has been reported that Bach1 is newly biosynthesized, possibly on ribosomes in the cytosol, and then transported into the nucleus [27]. We also demonstrated that the level of nuclear Bach1 protein 12 h after glycerol treatment exceeded the basal levels. Taken together, our findings suggest that the increase in cytosolic Bach1 protein expression after glycerol treatment may reflect *de novo* Bach1 protein synthesis, which compensates for the depletion of nuclear Bach1 protein. We may have failed to detect an increase in cytosolic Bach1 protein accompanying Bach1 nuclear export because Bach1 exported from the nucleus was reported to be rapidly degraded by the proteasome in cultured cells [27].

The pathophysiological significance of the dynamic changes in Bach1 expression in the nucleus and the cytosol is unclear. We do know that HO-1 plays a cytoprotective role in a rodent model of RM-AKI [2, 3], and the deletion of the Bach1 gene in mice confers protection against acute oxidative tissue injuries, including those to the cardiovascular system, respiratory tract, digestive tract, liver, and skin, which are mediated through the overexpression of HO-1 [28]. In contrast to beneficial effect of HO-1 induction in the acute phase of oxidative tissue injury, overexpression of HO-1 might have deleterious effects during the late phase of oxidative tissue injury after excess amounts of free heme have been catabolized. For example, HO-1 cleaves the heme moiety of hemoproteins and releases labile iron, which catalyzes the synthesis of free radicals [15, 29]. In addition, HO-1 overexpression might lead to a disruption of energy production rather than protection against oxidative stress, because several hemoproteins function in the mitochondria to mediate energy production [30]. Thus, the decrease in nuclear Bach1 protein expression and the increase in cytosolic Bach1 protein expression may contribute to the maintenance of the physiological milieu associated with heme metabolism, at least in part, and is mediated through the induction of HO-1 and the restoration of HO-1 expression to basal levels, possibly via the intracellular redistribution of Bach1 protein. Consistent with our notion, we previously demonstrated that the activation of Bach1 gene expression occurs in a rat model of carbon tetrachloride-induced oxidative hepatic injury, which can be attributed to free heme, a pro-oxidant released from hepatic cytochrome P450 [31]. Of course, further mechanistic analysis is clearly needed to substantiate our hypothesis since our description about Bach1 dynamics in the nucleus and cytosol is phenomenological.

In conclusion, for the first time to our knowledge, we demonstrate the dynamic changes in Bach1 expression in a rat model of RM-AKI. Our findings suggest that the increase in the concentration of intracellular free heme contributes to the pathogenesis of RM-AKI.

## References

[1] Boutaud O, Roberts LJ 2nd. Mechanism-based therapeutic approaches to rhabdomyolysis-induced renal failure. Free Radic Biol Med. 2011; 51: 1062–7. doi: 10.1016/j.freeradbiomed.2010.10.704 21034813 10.1016/j.freeradbiomed.2010.10.704 PMC3116013

[2] Nath KA, Balla G, Vercellotti GM, Balla J, Jacob HS, Levitt MD, et al Induction of heme oxygenase is a rapid, protective response in rhabdomyolysis in the rat. J Clin Invest. 1992; 90: 267–70. doi: 10.1172/JCI115847 1634613 10.1172/JCI115847 PMC443091

[3] Nath KA, Haggard JJ, Croatt AJ, Grande JP, Poss KD, Alam J. The indispensability of heme oxygenase-1 in protecting against acute heme protein-induced toxicity in vivo. Am J Pathol. 2000; 156: 1527–35. doi: 10.1016/S0002-9440(10)65024-9 10793064 10.1016/S0002-9440(10)65024-9 PMC1876926

[4] Sun J, Hoshino H, Takaku K, Nakajima O, Muto A, Suzuki H, et al Hemoprotein Bach1 regulates enhancer availability of heme oxygenase-1 gene. EMBO J. 2002; 21: 5216–24. doi: 10.1093/emboj/cdf516 12356737 10.1093/emboj/cdf516 PMC129038

[5] Suzuki H, Tashiro S, Hira S, Sun J, Yamazaki C, Zenke Y, et al Heme regulates gene expression by triggering Crm1-dependent nuclear export of Bach1. EMBO J. 2004; 23: 2544–53. doi: 10.1038/sj.emboj.7600248 15175654 10.1038/sj.emboj.7600248 PMC449764

[6] Igarashi K, Sun J. The heme-Bach1 pathway in the regulation of oxidative stress response and erythroid differentiation. Antioxid Redox Signal. 2006; 8: 107–18. doi: 10.1089/ars.2006.8.107 16487043 10.1089/ars.2006.8.107

[7] Ozono R. New biotechnological methods to reduce oxidative stress in the cardiovascular system: focusing on the Bach1/heme oxygenase-1 pathway. Curr Pharm Biotechnol. 2006; 7: 87–93. 16724942 10.2174/138920106776597630

[8] Shibahara S, Müller R, Taguchi H, Yoshida T. Cloning and expression of cDNA for rat heme oxygenase. Proc Natl Acad Sci U S A. 1985; 82: 7865–9. 3865203 10.1073/pnas.82.23.7865 PMC390870

[9] Yamamoto M, Kure S, Engel JD, Hiraga K. Structure, turnover, and heme-mediated suppression of the level of mRNA encoding rat liver delta-aminolevulinate synthase. J Biol Chem. 1988; 263: 15973–9. 3182776

[10] Kitamuro T, Takahashi K, Ogawa K, Udono-Fujimori R, Takeda K, Furuyama K, et al Bach1 functions as a hypoxia-inducible repressor for the heme oxygenase-1 gene in human cells. J Biol Chem. 2003; 278: 9125–33. doi: 10.1074/jbc.M209939200 12511571 10.1074/jbc.M209939200

[11] Maeshima K, Takahashi T, Nakahira K, Shimizu H, Fujii H, Katayama H, et al A protective role of interleukin 11 on hepatic injury in acute endotoxemia. Shock. 2004;21: 134–8. doi: 10.1097/01.shk.0000103386.98235.f6 14752286 10.1097/01.shk.0000103386.98235.f6

[12] Nakahira K, Takahashi T, Shimizu H, Maeshima K, Uehara K, Fujii H, et al Protective role of heme oxygenase-1 induction in carbon tetrachloride-induced hepatotoxicity. Biochem Pharmacol. 2003; 66: 1091–105. 12963497 10.1016/s0006-2952(03)00444-1

[13] Shah SV, Walker PD. Evidence suggesting a role for hydroxyl radical in glycerol-induced acute renal failure. Am J Physiol. 1988; 255: 438–43. 10.1152/ajprenal.1988.255.3.F438 2843051

[14] Maines MD. Heme oxygenase: function, multiplicity, regulatory mechanisms, and clinical applications. FASEB J. 1988; 2: 2557–68. 3290025

[15] Nath KA. Heme oxygenase-1: a provenance for cytoprotective pathways in the kidney and other tissues. Kidney Int. 2006; 70: 432–43. doi: 10.1038/sj.ki.5001565 16775600 10.1038/sj.ki.5001565

[16] Alam J, Shibahara S, Smith A. Transcriptional activation of the heme oxygenase gene by heme and cadmium in mouse hepatoma cells. J Biol Chem. 1989; 264:6371–5. 2703493

[17] Fujita H, Sassa S. The rapid and decremental change in haem oxygenase mRNA during erythroid differentiation of murine erythroleukaemia cells. Br J Haematol. 1989; 73: 557–60. 2611141 10.1111/j.1365-2141.1989.tb00297.x

[18] Iwasa F, Sassa S, Kappas A. Delta-Aminolaevulinate synthase in human HepG2 hepatoma cells. Repression by haemin and induction by chemicals. Biochem J. 1989; 262: 807–13. 2556111 10.1042/bj2620807 PMC1133345

[19] Shimizu H, Takahashi T, Suzuki T, Yamasaki A, Fujiwara T, Odaka Y, et al Protective effect of heme oxygenase induction in ischemic acute renal failure. Crit Care Med. 2000; 28: 809–17. 10752834 10.1097/00003246-200003000-00033

[20] Maeshima K, Takahashi T, Uehara K, Shimizu H, Omori E, Yokoyama M, et al Prevention of hemorrhagic shock-induced lung injury by heme arginate treatment in rats. Biochem Pharmacol. 2005 6 1; 69(11): 1667–80. doi: 10.1016/j.bcp.2005.03.007 15896346 10.1016/j.bcp.2005.03.007

[21] Inoue K, Takahashi T, Uehara K, Shimizu H, Ido K, Morimatsu H, et al Protective role of heme oxygenase 1 in the intestinal tissue injury in hemorrhagic shock in rats. Shock. 2008 2; 29(2): 252–61. doi: 10.1097/shk.0b013e3180cab913 17693937 10.1097/shk.0b013e3180cab913

[22] Sassa Shigeru. Biological implications of Heme Metabolism. J Clin Biohem Nutr. 2006; 38: 138–155.

[23] Gburek J, Birn H, Verroust PJ, Goj B, Jacobsen C, Moestrup SK, et al Renal uptake of myoglobin is mediated by the endocytic receptors megalin and cubilin. Am J Physiol Renal Physiol. 2003; 285: F451–8. doi: 10.1152/ajprenal.00062.2003 12724130 10.1152/ajprenal.00062.2003

[24] Suzuki H, Tashiro S, Sun J, Doi H, Satomi S, Igarashi K. Cadmium induces nuclear export of Bach1, a transcriptional repressor of heme oxygenase-1 gene. J Biol Chem. 2003; 278: 49246–53. doi: 10.1074/jbc.M306764200 14504288 10.1074/jbc.M306764200

[25] Ogawa K, Sun J, Taketani S, Nakajima O, Nishitani C, Sassa S, et al Heme mediates derepression of Maf recognition element through direct binding to transcription repressor Bach1. EMBO J. 2001; 20: 2835–43. doi: 10.1093/emboj/20.11.2835 11387216 10.1093/emboj/20.11.2835 PMC125477

[26] Raval CM, Zhong JL, Mitchell SA, Tyrrell RM. The role of Bach1 in ultraviolet A-mediated human heme oxygenase 1 regulation in human skin fibroblasts. Free Radic Biol Med. 2012; 52: 227–36. doi: 10.1016/j.freeradbiomed.2011.10.494 22107958 10.1016/j.freeradbiomed.2011.10.494

[27] Kaspar JW, Jaiswal AK. Antioxidant-induced phosphorylation of tyrosine 486 leads to rapid nuclear export of Bach1 that allows Nrf2 to bind to the antioxidant response element and activate defensive gene expression. J Biol Chem. 2010; 285: 153–62. doi: 10.1074/jbc.M109.040022 19897490 10.1074/jbc.M109.040022 PMC2804160

[28] Igarashi K, Watanabe-Matsui M. Wearing red for signaling: the heme-bach axis in heme metabolism, oxidative stress response and iron immunology. Tohoku J Exp Med. 2014; 232: 229–53. 24681888 10.1620/tjem.232.229

[29] Ryter SW, Tyrrell RM. The heme synthesis and degradation pathways: role in oxidant sensitivity. Heme oxygenase has both pro- and antioxidant properties. Free Radic Biol Med. 2000; 28: 289–309. 11281297 10.1016/s0891-5849(99)00223-3

[30] Okada S, Muto A, Ogawa E, Nakanome A, Katoh Y, Ikawa S, et al Bach1-dependent and -independent regulation of heme oxygenase-1 in keratinocytes. J Biol Chem. 2010; 285: 23581–9. doi: 10.1074/jbc.M109.068197 20501657 10.1074/jbc.M109.068197 PMC2911280

[31] Tanioka N, Shimizu H, Takahashi T, Omori E, Kuroda K, Shibata M, et al Induction of hepatic Bach1 mRNA expression by carbon tetrachloride-induced acute liver injury in rats. Biomed Rep. 2014; 2: 359–363. doi: 10.3892/br.2014.235 24748974 10.3892/br.2014.235 PMC3990211

